# Balancing Non-Equilibrium Driving with Nucleotide Selectivity at Kinetic Checkpoints in Polymerase Fidelity Control

**DOI:** 10.3390/e20040306

**Published:** 2018-04-23

**Authors:** Chunhong Long, Jin Yu

**Affiliations:** Beijing Computational Science Research Center, Beijing 100193, China

**Keywords:** polymerase, non-equilibrium, kinetic checkpoint, nucleotide selection, fidelity control

## Abstract

High fidelity gene transcription and replication require kinetic discrimination of nucleotide substrate species by RNA and DNA polymerases under chemical non-equilibrium conditions. It is known that sufficiently large free energy driving force is needed for each polymerization or elongation cycle to maintain far-from-equilibrium to achieve low error rates. Considering that each cycle consists of multiple kinetic steps with different transition rates, one expects that the kinetic modulations by polymerases are not evenly conducted at each step. We show that accelerations at different kinetic steps impact quite differently to the overall elongation characteristics. In particular, for forward transitions that discriminate cognate and non-cognate nucleotide species to serve as kinetic selection checkpoints, the transition cannot be accelerated too quickly nor retained too slowly to obtain low error rates, as balancing is needed between the nucleotide selectivity and the non-equilibrium driving. Such a balance is not the same as the speed-accuracy tradeoff in which high accuracy is always obtained at sacrifice of speed. For illustration purposes, we used three-state and five-state models of nucleotide addition in the polymerase elongation and show how the non-equilibrium steady state characteristics change upon variations on stepwise forward or backward kinetics. Notably, by using the multi-step elongation schemes and parameters from T7 RNA polymerase transcription elongation, we demonstrate that individual transitions serving as selection checkpoints need to proceed at moderate rates in order to sustain the necessary non-equilibrium drives as well as to allow nucleotide selections for an optimal error control. We also illustrate why rate-limiting conformational transitions of the enzyme likely play a significant role in the error reduction.

## 1. Introduction

Template-based polymerization is fundamental to processes such as gene replication and transcription. During the polymerization processes, protein enzymes such as polymerases translocate along DNA or RNA and use one chain on the track as the template to synthesize a new chain of nucleic acid (NA), based on Watson-Crick base pairing. Basically, the polymerization can happen without the enzyme at very low speeds and with low fidelity. The enzymes essentially accelerate the polymerization chemical cycles, and often significantly improve the fidelity [1,2,3,4,5]. No matter whether it is under the enzyme catalysis or not, continuous polymerization or elongation process needs to be supported by the chemical potentials of the reactants over that of the products, so that the growth of the polymer chain is sustained under chemical non-equilibrium [6,7,8]. How the polymerase enzyme modulates the kinetics of the intermediate states within each nucleotide addition cycle (NAC) has been a central issue to be understood. 

It has been well shown that the high fidelity of template-based polymerization is often achieved at the far-from-equilibrium condition, when the driving force from the chemical free energy is strong [6,9]. Meanwhile, it has been pointed out that at a close-to-equilibrium condition, when the chemical driving force is very low, there exists an entropy-driven regime in which the growth of the synthesizing polymer chain is supported by the incorporation of erroneous substrate species [6]. Only when the chemical free energy force rises sufficiently can the error rate decrease and finally converge to a low value at the fast growth condition.

Indeed, the connection of a fast growth of the synthesizing polymer chain with a low error rate also reflects the key feature of kinetic discrimination, in which high accuracy is achieved at a high speed [6,10]. It had been noticed that the free energy differences between incorporating a cognate (right) nucleotide and a non-cognate (wrong) one are generally small under solution conditions (e.g., less than ~3 or 4 k_B_T), and can only supports error rates larger than ~10^−2^ at close to equilibrium conditions. Yet except for some error-prone polymerases, surprisingly low error rates (e.g., 10^−4^~10^−9^) could be achieved physiologically under polymerase actions in the gene replication or transcription processes [2,3]. Aside from proofreading steps that remove erroneous nucleotides after catalysis to quench the error rate for about one to three orders of magnitude, multiple kinetic steps commonly exist prior to the catalysis reaction to repetitively select against the non-cognate nucleotide incorporation [11,12,13]. 

The idea of multi-step checkpoints had already been embedded in the kinetic proofreading framework suggested early on by Hopfield and Ninio [14,15]. Stepwise kinetics in the enzymatic cycle of polymerization process have also been dissected experimentally in recent years [11,12]. Nevertheless, investigations on multi-step kinetics for the polymerization fidelity control have not been sufficiently conducted. Ideally, a generic two-state cycling model of the polymerization, with reactant and product states connected by forward and backward rates, seems to provide sufficient insights on the polymer growth at the far-from-equilibrium conditions [6], or on fidelity control with energetic or kinetic discrimination [10]. Nevertheless, by including additional kinetic steps and intermediate states into consideration, one would be able to separate the substrate association from the subsequent catalysis, at least, or decouple the solution concentration impacts from the protein conformational transition effects. In particular, the RNA polymerases (RNAPs) have been suggested to work as Brownian motors ratcheting along DNA [16,17,18,19], in which NTP (nucleotide triphosphate) binding and addition serve as a “paw” to prevent backward translocation or sliding of the RNAP on DNA. By separating the translocation step from the NTP incorporation kinetics, one would also be able to demonstrate a “selective ratcheting” mechanism, in which only the cognate substrate incorporation allows successful ratcheting while the non-cognate species hinders the process. Basically, via the multi-step NAC, one can probe how exactly the internal kinetics of enzyme impacts on the overall elongation characteristics.

In this work, we investigated how polymerases achieve high fidelity in the three-state and five-state representations of the NAC. By modulating transition rates at different steps throughout the cycle, we examined how elongation and error rates along with other non-equilibrium thermodynamics characteristics are affected. It is revealed that the accelerations at different steps bring quite different impacts to overall rates and characteristics. In particular, for transitions serving as the kinetic checkpoints where the cognate/right and the non-cognate/wrong nucleotide substrates are differentiated, the transition rates cannot be either too small or too large in order to balance between the non-equilibrium driving and selectivity. Indeed, the three-state model that includes the reactant (post-translocation) state, the substrate (nucleotide triphosphate or NTP bound) state, and the product (pre-translocation) state, provides a minimal representation of the NAC with both the non-checkpoint and checkpoint transitions present. In the three-state model, the NTP binding is directly followed by the catalysis, then with a recovering transition to allow the polymerase to translocate one periodic or nucleotide (nt) distance to initiate for a next NAC. Accordingly, upon NTP binding to the substrate state, the non-cognate species can be either rejected via unbinding, or inhibited through catalysis, so that the transitions from the substrate state back to the reactant state and forward to the product state both serve as the kinetic checkpoints. In the five-state model, there exist two NTP association states, a pre-insertion state and a followed insertion/substrate state, as being identified in bacteriophage T7 RNAP or DNA polymerase (DNAP) systems [4,12,20,21]. Accordingly, there can be four transitions, both the backward and forward transitions from the respective pre-insertion and insertion states, serving for the kinetic checkpoints. We therefore were able to survey kinetic impacts from the additional checkpoints and make comparisons. 

The transcription elongation kinetics of the single-subunit viral T7 RNAP had been previously determined from both biochemical studies [21] and single molecule force measurements [17]. Hence, we were able to analyze fidelity control in the representative T7 RNAP system, using realistic kinetic parameters [22]. Nevertheless, we only address generically how the stepwise transition rates impact on the overall elongation characteristics. More recent measurements on the T7 RNAP activities have been conducted for both the cognate and non-cognate nucleotide species [23,24], and provide the basis for further determining the step-by-step nucleotide selection kinetics or energetics.

Below we present the NAC model construction, notations, and then show the results on the speed and fidelity control, together with the other elongation characteristics including entropy production and energetic expenditure, all in the non-equilibrium steady state (NESS) context.

## 2. Methods

### 2.1. A Three-State Representation of the NAC with Selection 

**Figure 1:**
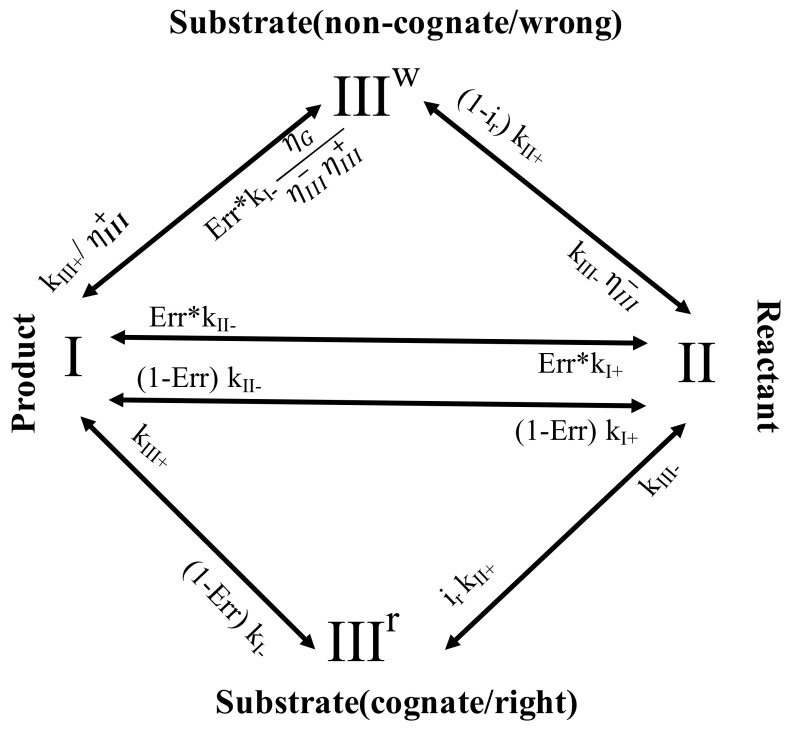
The kinetic scheme for the three-state nucleotide addition cycle (NAC) with selection. Since the cognate (right) and non-cognate (wrong) nucleotide species are differentiated in the substrate state *III*, one splits the cycle into two pathways for the right and wrong substrate species. Correspondingly, one has a population vector $\Pi ={\left(P_{I},P_{II},P_{III}^{r},P_{III}^{w}\right)}^{T}$ to describe the overall state probability distributions.

A three-state NAC with selection model was constructed as shown in Figure 1. The model starts from a product state (*I*, pre-translocation), and recovers to a reactant state (*II*, post-translocation), during which the polymerase is allowed to translocate along the NA track for one periodic length (one nucleotide or nt), with forward and backward rates as $k_{I+}$ and $k_{II-}$, respectively. 

Then, an incoming NTP binds to the enzyme into a substrate state (*III*), which can essentially differentiate the nucleotide species and select against the wrong ones, either by rejection or inhibition. In the rejection, the rate of the backward transition (*III* → *II*), or the substrate unbinding/off rate, is enhanced for the wrong species over that of the right ones ($k_{III-}^{w}>k_{III-}^{r}$), with a selection strength defined as ηIII−=kIII−wkIII−r (>>1 for strong selection); In the inhibition, the rate of the forward transition (*III* → *I*), or the catalysis reaction rate, is reduced for the wrong species, comparing to that of the right ones ($k_{III+}^{w}<k_{III+}^{r}$), and the selection strength is defined as ηIII+=kIII+rkIII+w (>>1 for strong selection). Importantly, since the right and wrong nucleotide species are differentiated at the substrate state *III*, we separate the state *III* population into the right and wrong ones. Correspondingly, one uses a population vector $\Pi ={\left(P_{I},P_{II},P_{III}^{r},P_{III}^{w}\right)}^{T}$ to describe the overall state probability distributions, with the inter-state transitions characterized by the chemical master equation:

(1) $$\frac{d}{dt}\Pi =M\Pi$$

where M is a 4 × 4 transition rate matrix:

$$\left\{\begin{array}{cccc} -(1-Err)k_{1-}-Err\cdot k_{1-}\frac{\eta_{G}}{\eta_{III}^{-}\eta_{III}^{+}}-k_{1+} & k_{II-} & k_{III+} & \frac{k_{III+}}{\eta_{III}^{+}}\\ k_{1+} & -k_{II-}-k_{II+} & k_{III-} & k_{III-}\eta_{III}^{-}\\ -(1-Err)k_{1-} & i_{r}k_{II+} & -k_{III-}-k_{III+} & 0\\ Err\cdot k_{1-}\frac{\eta_{G}}{\eta_{III}^{-}\eta_{III}^{+}} & (1-i_{r})k_{II+} & 0 & -k_{III-}\eta_{III}^{-}-\frac{k_{III+}}{\eta_{III}^{+}} \end{array}\right\}$$

By default, the rate constants *k_i_ ±* (*i* = *I*, *II* and *III*) without superscripts refer to that of the right substrate species. For the wrong substrate species, the kinetic rates (with the superscript *w*) are denoted either by the related selection strengths $\eta_{III}^{-}$ and $\eta_{III}^{+}$, as $k_{III-}^{w}=\eta_{III}^{-}k_{III-}$ and kIII+w=kIII+ηIII+ respectively, or kept the same (multiplied by a population factor 1-*i_r_* or *Err*, see the scheme in Figure 1, and *i_r_* and *Err* below) as that of the right species for transitions without differentiation (*I* ↔ *II*, and *I*/*II* → *III*). Consequently, the populations and fluxes of the right or wrong species are treated separately. For the substrate binding transition (*II* → *III*), suppose that the input right NTP accounts for a portion of *i_r_* (<1) from solution, at the entry to the polymerase active site, the forward transition probability fluxes are then written as $i_{r}k_{II+}P_{II}$ and $\left(1-i_{r}\right)k_{II+}P_{II}$ for the right and wrong species, respectively. Correspondingly, the respective fluxes for the right and wrong species between states *II* and *III* are $J^{r}=i_{r}k_{II+}P_{II}-k_{III-}P_{III}^{r}$ and $J^{w}=\left(1-i_{r}\right)k_{II+}P_{II}-\eta_{III}^{-}k_{III-}P_{III}^{w}$. The error rate can then be defined as the ratio between the wrong and the total fluxes $Err=J^{w}/J$, with $J=J^{r}+J^{w}$ being the total flux. At the steady state, the state populations do not change, so that the net flux keeps constant throughout the respective NACs of the right and wrong species. One thus obtains the steady-state solution of Equation (1) by solving:

(2) MΠ=0

(3) $$Err=\frac{(1-i_{r})k_{II+}P_{II}-\eta_{III}^{-}k_{III-}P_{III}^{w}}{P_{I}k_{I+}-P_{II}k_{II-}}$$

In addition, one needs to take into account that without the enzyme, the free energy difference between incorporating the right and wrong substrate species is—δ_G_ (with δ_G_ > 0 and being small; we used δ_G_ ~ 2 k_B_T by default). The free energy difference should not be altered due to the enzymatic reaction, hence, one needs to set a constraint on the relative stability of the incorporated wrong substrate to the right one in the product state, by resetting the reversal catalytic rate of the wrong species (*III*→*I*) to $\frac{\eta_{G}}{\eta_{III}^{-}\eta_{III}^{+}}k_{I-}$ and the corresponding backward flux as $Err\frac{\eta_{G}}{\eta_{III}^{-}\eta_{III}^{+}}k_{I-}P_{I}$, where $\eta_{G}=e^{\delta_{G}}$.

The exact solution of Equations (2) and (3) can then be obtained. In the far-from-equilibrium case when the catalytic step of the NAC approaches to be irreversible, that is, $k_{I-}\to 0$, one can obtain a simplified expression of the error rate as:

(4) $$Err=\frac{1-i_{r}}{1+i_{r}\frac{k_{III-}}{k_{III+}+k_{III-}}\left(\eta_{III}^{-}\eta_{III}^{+}-1\right)}$$

### 2.2. A Five-State Representation of the NAC with Selection 

**Figure 2:**
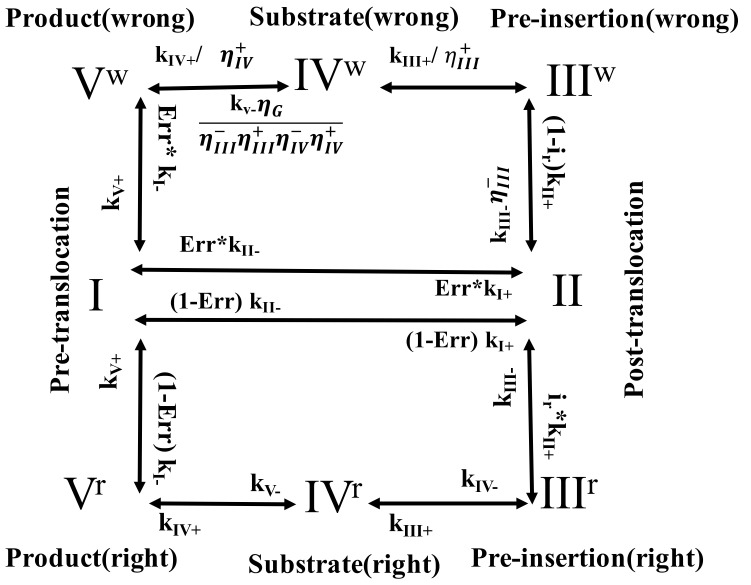
The kinetic scheme for the five-state NAC with selection. The cognate (right) and non-cognate (wrong) nucleotide species are differentiated into two NAC pathways, and one uses a population vector $II={\left(P_{I},P_{II},P_{III}^{r},P_{III}^{w},P_{IV}^{r},P_{IV}^{w},P_{V}^{r},P_{V}^{w}\right)}^{T}$ to describe the state probability distributions.

Then, we used a slightly more detailed kinetic scheme with five states, e.g., as for T7 RNAP [20,21,25], to describe the NAC or the RNAP elongation cycle (see Figure 2). Essentially, there are two NTP association states, the pre-insertion state *III* and the insertion/substrate state *IV*, which differentiate the right and wrong nucleotide species in the five-state representation.

Correspondingly, the first selection checkpoint (*III* → *II*) rejects wrong nucleotides immediately upon the NTP binding to the pre-insertion state (at the strength ηIII−=kIII−wkIII−r), which is similar to that in the three-state scheme. The next selection checkpoint (*III* → *IV*) then inhibits wrong nucleotides from inserting into the active site (ηIII+=kIII+rkIII+w). The third selection checkpoint (*IV* → *III*) destabilizes the wrong nucleotides after being inserted at the substrate state *IV* (ηIV−≡kIV−wkIV−r). The last selection checkpoint (*IV* → *V*) inhibits catalytic reaction of the wrong nucleotides (ηIV+≡kIV+rkIV+w). The transition between the product state *V* and the pre-translocation state *I* then represents the product PPi dissociation step, which is not explicitly modeled in the three-state scheme as the product and pre-translocation state are treated identical in that case. 

Since the right and wrong nucleotide species are accordingly split into two NAC pathways (see Figure 2), a population vector $II={\left(P_{I},P_{II},P_{III}^{r},P_{III}^{w},P_{IV}^{r},P_{IV}^{w},P_{V}^{r},P_{V}^{w}\right)}^{T}$ is used to describe the overall state probability distributions, and the corresponding master equation is:

(5) $$\frac{d}{\mathrm{dt}}II=MII$$

where *M* is now a 8 × 8 transition rate matrix as:

$$\left\{\begin{array}{cccccccc} -k_{I-}-k_{I+} & k_{II-} & 0 & 0 & 0 & 0 & k_{V+} & k_{V+}\\ k_{I+} & -k_{II-}-k_{II+} & k_{III-} & k_{III-}\cdot \eta_{III}^{-} & 0 & 0 & 0 & 0\\ 0 & i_{r}k_{II+} & -k_{III-}-k_{III+} & 0 & k_{IV-} & 0 & 0 & 0\\ 0 & (1-i_{r})k_{II+} & 0 & -k_{III-}\cdot \eta_{III}^{-}-\frac{k_{III+}}{\eta_{III}^{+}} & 0 & k_{IV-}\cdot \eta_{IV}^{-} & 0 & 0\\ 0 & 0 & k_{III+} & 0 & -k_{IV-}-k_{IV+} & 0 & k_{V-} & 0\\ 0 & 0 & 0 & \frac{k_{III+}}{\eta_{III}^{+}} & 0 & -k_{IV-}\cdot \eta_{IV}^{-}-\frac{k_{IV+}}{\eta_{IV}^{+}} & 0 & \frac{k_{V-}\cdot \eta_{G}}{\eta_{III}^{-}\eta_{III}^{+}\eta_{IV}^{-}\eta_{IV}^{+}}\\ (1-Err)k_{I-} & 0 & 0 & 0 & k_{IV+} & 0 & -k_{V-}-k_{V+} & 0\\ Err\cdot k_{I-} & 0 & 0 & 0 & 0 & \frac{k_{IV+}}{\eta_{IV}^{+}} & 0 & -k_{V+}-\frac{k_{V-}\cdot \eta_{G}}{\eta_{III}^{-}\eta_{III}^{+}\eta_{IV}^{-}\eta_{IV}^{+}} \end{array}\right\}$$

One can then obtains the steady state solutions of Equation (5) similarly as for the three-state scheme.

### 2.3. The Entropy Production, Heat Dissipation or Free Energy Expenditure in the NESS

For the above three-state or five-state of NAC with a selection model at NESS, one can calculate for each cycle the entropy production $\Xi_{p}$, the heat dissipation *H_d_* (or the free energy expenditure), and the total entropy change $\Xi_{t}=\Xi_{p}-H_{d}$ [26]. The entropy production rate ${\dot{\Xi }}_{p}$ is defined according to the probability fluxes as ${\dot{\Xi }}_{p}=k_{B}\sum_{i=1}^{N}\left(J_{i,i+1}-J_{i+1,i}\right)ln\left(J_{i,i+1}/J_{i+1,i}\right)$ with *i*, *i* + *1* representing two consecutive discrete states in the enzymatic cycle (for *N* kinetic states, *N* + *1* resets to 1). Meanwhile, using the steady-state flux *J*, the heat dissipation rate is written as ${\dot{H}}_{d}=Jk_{B}T\sum_{i=1}^{N}ln\frac{k_{i+}}{k_{i+1-}}$. When there are both right and wrong substrate species, one can write down ${\dot{\Xi }}_{p}$ for the right and wrong species as ${\dot{\Xi }}_{p}^{r}=J\left(1-Err\right)\left[\Delta G_{c}^{r}/T-k_{B}\ln\left(1-Err\right)\right]$ and ${\dot{\Xi }}_{p}^{w}=J\cdot Err\cdot \left(\Delta G_{c}^{w}/T-k_{B}lnErr\right)$, where $\Delta G_{c}^{r}$ and $\Delta G_{c}^{w}$ denote the free energy inputs for the right and wrong substrates, respectively. Additionally, one needs to take into account the solution concentration and stability disparities between the right and wrong nucleotide species, so that one has $\Delta G_{c}^{r}=\Delta G_{c}+k_{B}Tlni_{r}$ and $\Delta G_{c}^{w}=\Delta G_{c}+k_{B}Tln\left(1-i_{r}\right)-\delta_{G}$ with $\Delta G_{c}=k_{B}T\sum_{i=1}^{N}ln\frac{k_{i+1}}{k_{i+1-}}$ being the common or ‘standard’ part. The total entropy production rate is then counted as:

(6) $${\dot{\Xi }}_{p}={\dot{\Xi }}_{p}^{r}+{\dot{\Xi }}_{p}^{w}=J\left[\Delta G_{c}/T+\left(1-Err\right)k_{B}ln\frac{i_{r}}{1-Err}+Err\cdot \left(k_{B}ln\frac{1-i_{r}}{Err}-\delta_{G}/T\right)\right]$$

When *Err* → 0, ${\dot{\Xi }}_{p}$ converges to $J\Delta G_{c}^{r}/T$. For each NAC, the entropy production per cycle is then counted as $\Xi_{p}={\dot{\Xi }}_{p}/J$. Note that ${\dot{\Xi }}_{p}\geq 0$ always holds [26]. Meanwhile, the heat dissipation rate ${\dot{H}}_{d}=J\left[\left(1-Err\right)\Delta G_{c}^{r}+Err\cdot \Delta G_{c}^{w}\right]$ becomes:

(7) $${\dot{H}}_{d}=J\left\{\Delta G_{c}+\left(1-Err\right)k_{B}Tlni_{r}+Err\cdot \left[k_{B}Tln\left(1-i_{r}\right)-\delta_{G}\right]\right\}$$

Similarly, $H_{d}={\dot{H}}_{d}/J$ represents the amount of heat dissipation for each NAC, which can be regarded as the free energy expenditure or the free energy driving force [6]. As a result the overall or net entropy change per NAC is:

(8) $${\dot{\Xi }}_{t}/J=\Xi_{p}-H_{d}/T=k_{B}[(1-Err)\ln\frac{1}{1-Err}+Err\ln\frac{1}{Err}]$$

with the first and second terms coming from the right and wrong substrate species, respectively. One can see that the net entropy change for each NAC in the template-based polymerization is only determined by the error rate, as only the right and wrong species are considered.

## 3. Results

Upon varying individual forward (*k*_*i*+_) or backward (*k*_*i*−_) rates while keeping the selection strength constant (e.g., $\eta_{i}^{\pm }=100$), we solved the steady-state equations for both the three-state and five-state models, and obtained the corresponding elongation rate (*J*) and the error rate (*Err*), along with the entropy production ($\Xi_{p}$ and its rate) and heat dissipation (*H_d_* and its rate). In the three-state model, we used kinetic parameters that reproduced the single molecule force measurements on T7 RNAP transcription elongation [17]; additional parameters in the five-state model were taken from transient state kinetics measurements [21,22].

### 3.1. Acceleration without Differentiation: Low Error Rate Is Achieved at Far-From-Equilibrium

First, we examined how the elongation characteristics change in the three-state scheme with an increase in the forward or NTP binding/on rate toward the substrate state *III* ($k_{II+}=k_{T}^{0}\left[NTP\right]$). In the calculation, we kept constant selection strength $\eta_{III}^{-}=\eta_{III}^{+}=100$ (from the kinetic bias against the non-cognate species at the selection checkpoint) and $\eta_{G}=10$ (from the energy bias against non-cognate incorporation at the end of NAC). By default, we used $k_{T}^{0}$~2 μM^−1^s^−1^ and $k_{p}^{0}$~1 μM^−1^s^−1^ for the binding of NTP and PPi, respectively, and set comparatively high concentrations of NTP and PPi (e.g., [NTP] = 588 μM and [PPi] = 100 μM).

**Figure 3:**
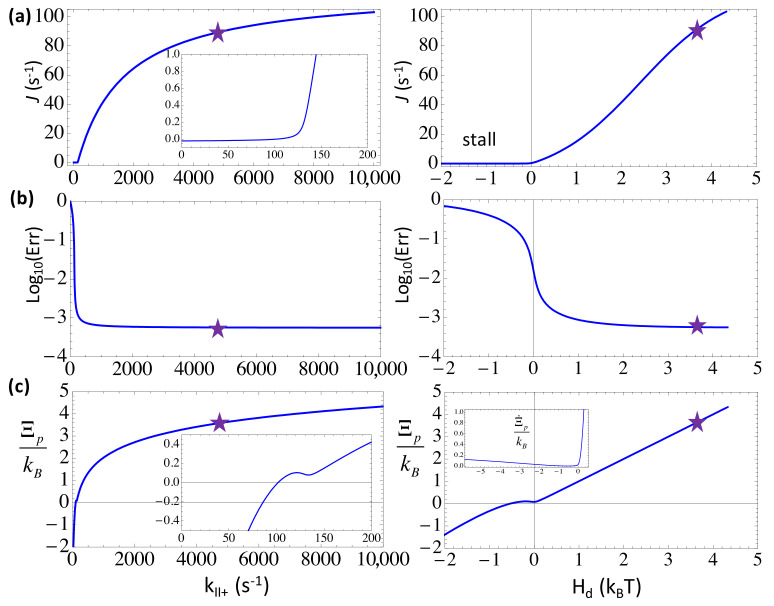
The elongation characteristics upon varying the substrate binding rate *k*_*II*+_ in the three-state scheme. The elongation rate *J* (**a**); the error rate *Err* in the logarithmic value (**b**); and the entropy production per cycle $\Xi_{p}$ (**c**) are shown versus *k*_*II*+_ (left) or versus the free energy expenditure *H_d_* (right). The corresponding characteristic values at the default value of *k*_*II*+_ = 4.7 × 10^3^ s^−1^ ([NTP] = 588 uM) are labeled by stars. Note that the entropy production rate ${\dot{\Xi }}_{p}$ is also shown in the inset of (**c**) right.

From Figure 3, one can see that a large value of *k*_*II*+_ (→ 10^4^ s^−1^) leads to a close to saturation elongation rate *J* (~100 s^−1^) and a minimum error rate *Err* (~6 × 10^−4^) at a far-from-equilibrium condition, with a sufficiently high heat dissipation *H_d_* (>4 k_B_T) or free energy expenditure to drive each NAC. Since the error rate is very small in the end, the incorporated nucleotide species are almost uniformly the cognate ones, the net entropy production accordingly approaches zero, and the entropy production $\Xi_{p}$ converges toward *H_d_*/T. The trend well indicates an enhanced non-equilibrium driving upon the accelerated substrate association until saturation. 

For example, a comparatively high [NTP] (588 μM) and a low [NTP] (50 μM) lead to high and low speeds (*J* ~ 70 s^−1^ and 17 s^−1^), respectively, while the error rates remain low for both cases (*Err* ~6 × 10^−4^ and 8 × 10^−4^), with the respective free energy expenditure at *H_d_* ~ 3.6 k_B_T and 1.1 k_B_T. Indeed, one sees that as long as *H_d_* > 1 k_B_T, or say, above the thermal fluctuation level, the error rate drops to a quite low value, while a high speed requires an even stronger free energy being driven, e.g., *H_d_* > 2~3 k_B_T.

The error rate rises by about one order of magnitude (*Err* ~ 10^−2^) from right below *H_d_* ~ 1 k_B_T toward a zero driving at *H_d_* ~ 0, as the elongation rate approaches zero. Note that for *H_d_* < 0, such as under an extremely low substrate concentration (e.g., [NTP] < 18 μM), the entropy ‘production’ drops to negative values, $\Xi_{p}<0$, except for the *H_d_* → 0-region. Nevertheless, the positive rate of the entropy production ${\dot{\Xi }}_{p}\geq 0$ is always maintained. One sees that the elongation rate *J* ~ 0 holds due to fairly strong selection against the non-cognate NTP species ($\eta_{III}^{-}=\eta_{III}^{+}=100$), which are abundant at this condition (*Err* ~ 10^−1^ with 75% non-cognate species from solution). Without the selection, a negative free energy driving force (*H_d_* < 0) would induce a negative elongation speed (results not shown). For *H_d_* ≥ −0.4 k_B_T, however, $\Xi_{P}>0$, a small window of an entropy driven regime of elongation reveals, which is mainly supported by non-cognate NTP additions to the growing NA chain [6].

In comparison, one can also enhance the backward rate *k*_*I*−_ that dictates the reversal of the catalytic reaction or the product (PPi) rebinding to the active site. Since *k*_*I*−_ ∝ [PPi], increasing [PPi] from 0.1 to 100 μM (keep [NTP] = 588 μM), the elongation and error rates do not vary much (e.g., *J* ~ 95 s^−1^ to 89 s^−1^, *Err* ~ 5.5 × 10^−4^ to 5.7 × 10^−4^). With increasing *k*_*I*−_, the elongation rate would only drop for *k*_*I*−_ > 10^3^ s^−1^; at *k*_*I*−_ ~4 × 10^3^ s^−1^, *J* ~ 0, the free energy driving force *H_d_* becomes slightly negative, the error rate rises to ~10^−1^, and the system approaches close to equilibrium. Under regular conditions (e.g., for [PPi] from 0.1 to 100 μM, *H_d_* ranges from ~3 to 10 k_B_T), as long as the NTP substrate concentration remains high, the system can be well maintained at non-equilibrium, and the error reduction remains effective.

### 3.2. Balancing the Non-Equilibrium Driving with the Substrate Differentiation at the Forward Selection Checkpoint by Maintaining a Moderate Substrate Addition Rate 

We showed the steady-state characteristics upon varying the substrate addition or catalysis rate *k*_*III*+_, which dictates the forward transition from the substrate state *III* toward the product state *I* in the three-state presentation. A non-cognate or wrong NTP bound to the active site of the polymerase can be selected against by inhibition to slow down its addition to the growing chain, i.e., via a lowered forward rate as $k_{III+}^{w}=k_{III+}/\eta_{III}^{+}$ where we set $\eta_{III}^{+}=100(>>1)$. By default, *k*_*III*+_ ~ 132 s^−1^ [17], which sets a rate-limiting step of NAC in T7 RNAP, accordingly, $k_{III+}^{w}\sim 1.3\;s^{-1}$. Due to the rate-limiting character, when *k*_*III*+_ increases above ~10^3^ s^−1^, the elongation rate rises significantly (to 400 ~ 600 s^−1^). Nevertheless, the error rate would also rise significantly from below 10^−3^ to ~10^−2^, in agreement with the elongation rate increase. The trend actually shows an energetic regime of substrate differentiation [10]. Where *k*_*III*+_ decreases to 10 s^−1^ below, or *H_d_* drops below ~1 k_B_T, the error rate also rises as the elongation rate diminishes. Like in other cases, *Err* ~ 10^−1^ as *H_d_* is slightly negative, indicating the close-to-equilibrium error-prone tendency [6], which can then be suppressed by fast kinetics as *H_d_* recovers to 0 ~ 4 k_B_T.

**Figure 4:**
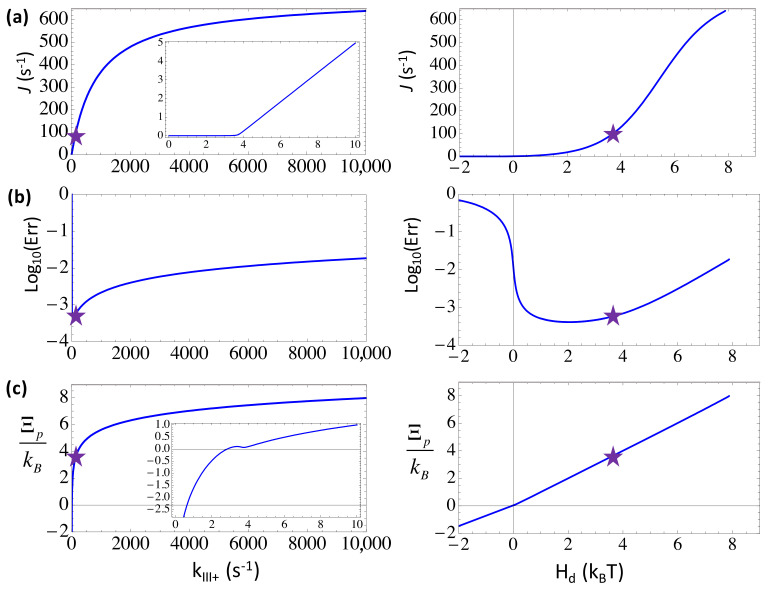
The elongation characteristics upon varying substrate addition or catalytic rate *k*_*III*+_ in the three-state scheme. The elongation rate *J* (**a**); the error rate *Err* in the logarithmic value (**b**); and the entropy production per cycle $\Xi_{p}$ (**c**) are shown versus *k*_*III*+_ (left) or versus the free energy expenditure *H_d_* (right). The corresponding characteristic values at the default value of *k*_*III*+_ = 132 s^−1^ are labeled by stars.

Interestingly, one sees that the system default value of *k*_*III*+_ ~ 132 s^−1^ (with *H_d_* ~ 3.6 k_B_T) allows the fastest nucleotide addition kinetic, beyond which the error rate would significantly rise (see Figure 4b). That is to say, even though an increase of *k*_*III*+_ to ~10^3^ s^−1^ or above significantly improves the overall elongation rate, the RNAP system has to avoid too fast substrate addition in order to allow sufficient differentiation. Meanwhile, slow kinetic on this rate-limiting step would not only drag down the overall elongation, but also lead to more errors at the close-to-equilibrium condition. Indeed, current system achieves a close-to-minimum error rate, ~6 × 10^−4^, at this seemingly optimal kinetic rate of addition ~10^2^ s^−1^.

### 3.3. Maintaining a Moderate Substrate Dissociation Rate to Coordinate with the Substrate Addition and Selection to Keep the Speed High 

**Figure 5:**
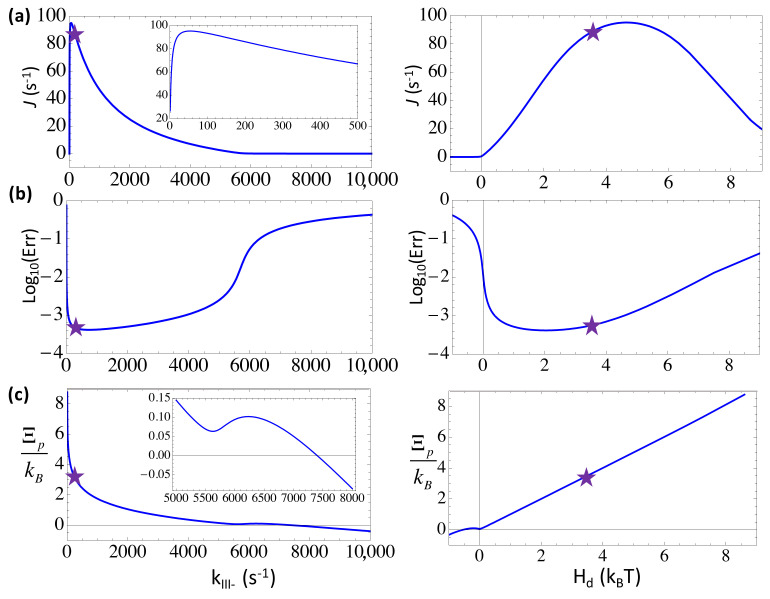
The elongation characteristics upon varying the substrate dissociation rate *k*_*III*−_ in the three-state scheme. The elongation rate *J* (**a**); the error rate *Err* in the logarithmic value (**b**); and the entropy production per cycle $\Xi_{p}$ (**c**) are shown versus *k*_*III*−_ (left) or versus the free energy expenditure *H_d_* (right). The corresponding characteristic values at the default value of *k*_*III*−_ = 160 s^−1^ are labeled by stars.

We also varied the substrate dissociation rate *k*_*III*−_ from the substrate-bound state *III* to obtain the corresponding steady state characteristics (see Figure 5). *k*_*III*−_ represents the rate of the substrate unbinding or the off rate, which serves as an initial screening to select against the non-cognate/wrong NTP species via an enhanced dissociation: $k_{III-}^{w}=\eta_{III}^{-}k_{III-}$, and we still $\eta_{III}^{-}=\eta_{III}^{+}=100$. For a dissociation constant *K*_M_ ~ 80 μM, we had *k*_*III*−_ ~ 160 s^−1^, so that $k_{III-}^{w}\sim 16,000s^{-1}$ significantly rejects the wrong NTP. Under the default setting, the error rate is already quite low (between 10^−4^~10^−3^) and the elongation rate is maintained to be high (~90 s^−1^). By increasing *k*_*III*−_ to ~10^3^ s^−1^, the error rate lowers slightly further (→10^−4^), while the elongation rate drops to about half the maximum value. Further increasing *k*_*III*−_ beyond 5000 s^−1^, the error rate also rises above 10^−3^ and the elongation rate almost diminishes (<2 s^−1^), as the system approaches to an equilibrium condition. On the other hand, if one lowers *k*_*III*−_ from ~10^2^ s^−1^ to 10 s^−1^ and below, one can observe a sharp drop of the elongation rate and a rise of the error rate. The error rate rise is due to the dissociation being too slow, which cannot efficiently reject the non-cognate substrate. In comparison, the elongation rate diminishes at *k*_*III*−_ → 0+ as *H_d_* increases > 5 k_B_T, which is quite different from other cases in which an increasing energetics leads to fast elongation. This happens because of simultaneously enforced selection ($\eta_{III}^{+}=100$) in the followed substrate addition transition; without the addition selection (i.e., $\eta_{III}^{+}=1$), the elongation rate would still reach above ~100 s^−1^ for a high *H_d_* (>5 k_B_T), and the error rate also rises as the rejection ($\eta_{III}^{-}=100$) does not work well for very low *k*_*III*−_ either. In brief, to maintain both a high speed and a low error rate in coordination with the subsequent selection in the nucleotide addition, one has to keep the substrate dissociation rate *k*_*III*−_ at a medium value (~10^2^ s^−1^) as well.

**Figure 6:**
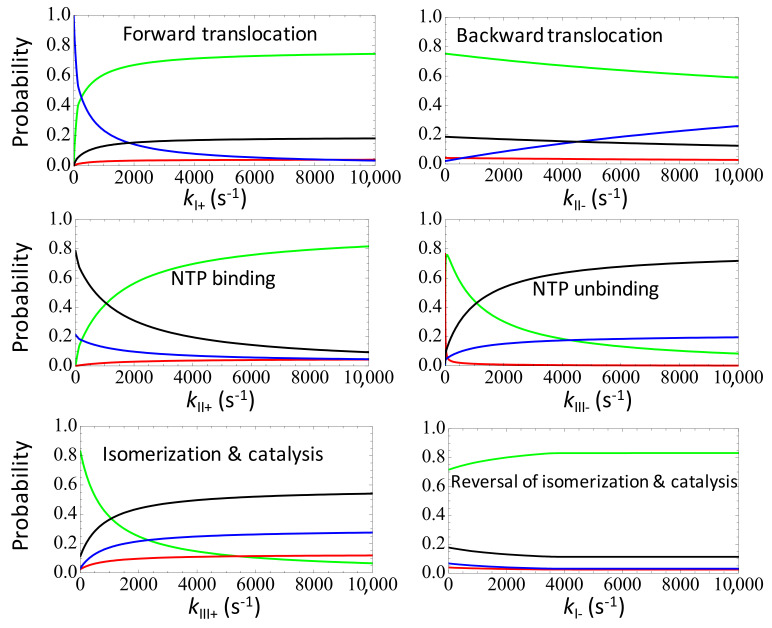
The non-equilibrium steady state (NESS) probability distributions upon varying individual rates in the three-state model of NAC with selection. The probabilities of the state *I*, *II*, *III^r^* and *III^w^* are shown in blue, black, green, and red curves. The left column shows the state population or probability changes upon the forward rate changes, while the right column shows that upon the backward rate changes.

Surveying the steady state properties by varying individual forward or backward rates in the three-state model of NAC with selection, one infers that in general, for the forward rate (*k*_*I*+_ or *k*_*II*+_) involving no differentiation between the right and wrong substrate species, a large rate leads to a high elongation rate and low error rate, as approaching to the far-from-equilibrium. Nevertheless, either the speed or error rate does not change further beyond saturation. In particular, for the current RNAP system, as the forward translocation rate *k*_*I*+_ > 1000 s^−1^, the error rate does not decrease further, while the convergence of the elongation rate to a maximum value requires *k*_*I*+_ > 2000 s^−1^. Note that the default translocation rate we used is *k*_*I*+_ ~ 5000 s^−1^. For the NTP binding rate, the error rate approaches to a minimum for *k*_*II*+_ > 400 s^−1^ ([NTP] > 50 μM), while the elongation rate approaches to saturation for *k*_*II*+_ > 2000 s^−1^ ([NTP] > 250 μM). The trend can also be revealed from the steady-state probability distributions upon individual rate variations (see Figure 6 left).

For the backward rates (*k*_*II*−_ or *k*_*I*−_) that are not involved in the nucleotide selection, the non-equilibrium is well achieved at small values of the rates: For the backward translocation, *k*_*II*−_ < 10^4^ s^−1^ appears to allow the far-from-equilibrium; for the PPi rebinding rate *k*_*I*−_, varying *k*_*I*−_ below 10^4^ s^−1^ almost brings no impacts on the steady state properties (see Figure 6 right). In contrast, for the NTP addition or catalytic rate *k*_*III*+_ that is reduced for the non-cognate substrate species, one can see that a high value of *k*_*III*+_ leads to a relatively high population of the wrong NTP bound state (*III^w^*) compared to that of the right substrate state (*III^r^*). Similarly, for the NTP unbinding rate *k*_*III*−_ that is enhanced for the wrong NTP species, either a large or a very small value of *k*_*III*−_ leads to comparable populations of the right and wrong substrate-bound states (*III^r^* and *III^w^*), which indicate comparatively high error rates.

### 3.4. The Rate-Limiting Conformational Transition of Polymerase Plays a Signficant Role in the Error Control

**Figure 7:**
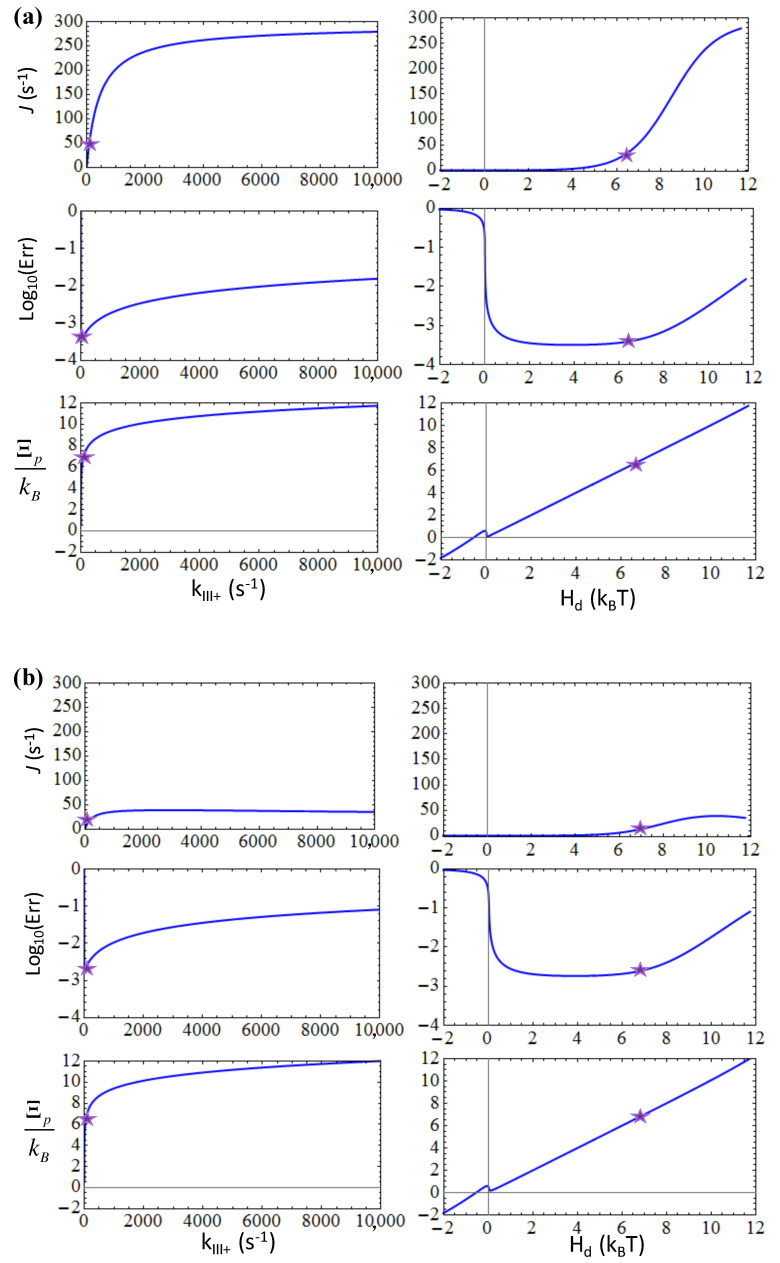
The elongation characteristics upon varying the insertion rate *k*_*III*+_ for the transition from the pre-insertion state (*III*) to the insertion state (*IV*) in the five-state scheme (see Figure 2). The elongation rate *J*, the error rate *Err* in the logarithmic value, and the entropy production per cycle $\Xi_{P}$ are shown versus *k*_*III*+_ or versus the free energy expenditure *H_d_* for: (**a**) The pre-insertion selection strategy ($\eta_{III}^{+}=\eta_{III}^{-}=100\;\&\;\eta_{IV}^{+}=\eta_{IV}^{-}=1$); (**b**) The post-insertion selection strategy ($\eta_{III}^{+}=\eta_{III}^{-}=1\;\&\;\eta_{IV}^{+}=\eta_{IV}^{-}=100$). The corresponding characteristic values at the default value of *k*_*III*+_ = 220 s^−1^ are labeled by stars.

**Figure 8:**
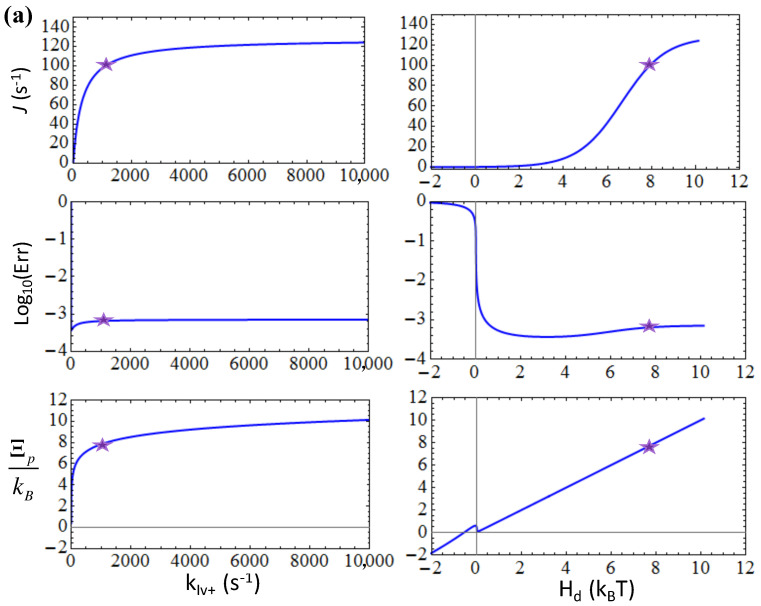

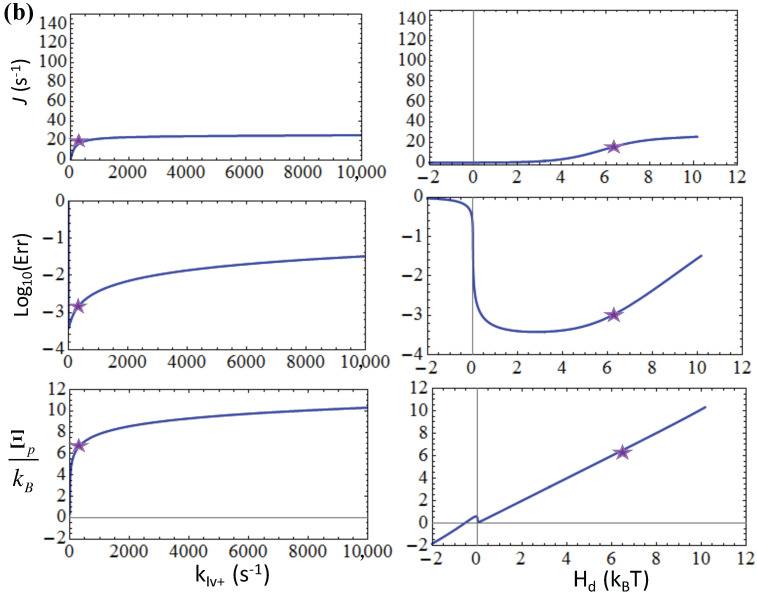
The elongation characteristics upon varying the post-insertion or catalytic rate *k*_*IV*+_ for the transition from the insertion state (*IV*) to the product state (*V*) in the five-state model. The elongation rate *J*, the error rate *Err* in the logarithmic value, and the entropy production per cycle $\Xi_{p}$ are shown versus *k*_*IV*+_ or versus the free energy expenditure *H_d_* for: (**a**) The pre-insertion selection strategy ($\eta_{III}^{+}=\eta_{III}^{-}=100\;\&\;\eta_{IV}^{+}=\eta_{IV}^{-}=1$); (**b**) The post-insertion selection strategy ($\eta_{III}^{+}=\eta_{III}^{-}=1\;\&\;\eta_{IV}^{+}=\eta_{IV}^{-}=100$). The corresponding characteristic values at the default value of *k*_*IV*+_ = 1000 s^−1^ are labeled by stars.

Then we expanded to the five-state NAC model with selection for T7 RNAP elongation. In the five-state representation, there are two states essentially involved in the substrate differentiation, the NTP pre-insertion (*III*) and insertion state (*IV*; see Figure 2). By default, we set the NTP insertion rate *k*_*III*+_ = 220 s^−1^ as experimentally measured [21]; as the catalytic rate *k*_*IV*+_ was too fast to be detected [21], we used *k*_*IV*+_ ~ 1000 s^−1^ as an estimation [22]. It had been reported that an average elongation error rate of T7 RNAP is ~10^−4^ [27]. Recent measurements on the fidelity control of single subunit RNAPs further pointed out the error rates down to 10^−5^ ~ 10^−6^ [23]. Nevertheless, it is still not clear which state, the pre-insertion or the insertion state, is mainly involved in the error control, or possibly whether the two play comparable roles together. To see how the variations of the insertion rate and catalytic rate impact on the overall elongation and error rates, we considered two selection strategies, one is the pre-insertion-dominated, while the other is the post-insertion-dominated. For the pre-insertion selection strategy that relies on the forward and backward transitions from the pre-insertion state *III* as *III* → *IV* and *II* → *II*, respectively, we set $\eta_{III}^{+}=\eta_{III}^{-}=100$ while keeping no selection thereafter ($\eta_{IV}^{+}=\eta_{IV}^{-}=1$). Similar to the three-state case, one finds that the forward transition rate *k*_*III*+_ that dictates the NTP insertion can neither be too small nor too large in order to keep the error rate low (see Figure 7a); again, current insertion rate *k*_*III*+_ ~ 220 s^−1^ corresponds well to a close-to-minimum error rate. In comparison, acceleration on the non-differentiating catalytic reaction at post-insertion, i.e., by increasing the catalytic rate *k*_*IV*+_, cannot impact much on the error rate, which remains below ~10^−3^ for *k*_*IV*+_ rising up to 10^4^ s^−1^ (see Figure 8a). Indeed, the current parameter setting under the pre-insertion selection strategy works well for the error control.

In the post-insertion selection strategy that relies on the forward and backward transitions from the insertion state *IV* as *IV* → *V* and *IV* → *III*, respectively, we then set $\eta_{III}^{+}=\eta_{III}^{-}=1$ and $\eta_{IV}^{+}=\eta_{IV}^{-}=100$. As expected, the involved forward transition rate or catalytic rate *k*_*IV*+_ cannot be too small or too large to keep the error rate low (see Figure 8b). It appears that the catalytic rate of ~1000 s^−1^ is actually already too large to maintain a low error rate < 10^−3^ (*Err* ~ 4 × 10^−3^ for *k*_*IV*+_ ~ 1000 s^−1^). Hence, the current parameter setting does not seem to be optimal for the post-insertion selection strategy. Unexpectedly, one notices that under the post-insertion selection strategy, the error rate would still increase significantly by increasing the insertion rate *k*_*III*+_ (see Figure 7b), even though the pre-insertion state *III* is not involved in the nucleotide differentiation under the post-insertion selection strategy.

In comparison, if one switches the rate-limiting event from the NTP insertion transition (*III* → *IV*) to the catalytic reaction (*IV* → *V*), e.g., by lowering the catalytic rate parameter to *k*_*IV*+_ < *k*_*III*+_ (e.g., setting *k*_*IV*+_ = 10 s^−1^ & keeping *k*_*III*+_ = 220 s^−1^) under the post-insertion selection strategy ($\eta_{III}^{+}=\eta_{III}^{-}=1\;\&\;\eta_{IV}^{+}=\eta_{IV}^{-}=100$), the increase of the pre-insertion rate *k*_*III*+_ would not impact much on the error rate (*Err* ~ 10^−3^ even when *k*_*III*+_ → 10^4^ s^−1^). Similarly, if we increased the NTP insertion rate parameter to *k*_*III*+_ > *k*_*IV*+_ (e.g., setting *k*_*III*+_ = 2200 s^−1^ & keeping *k*_*IV*+_ = 1000 s^−1^) under the pre-insertion selection strategy ($\eta_{III}^{+}=\eta_{III}^{-}=100\;\&\;\eta_{IV}^{+}=\eta_{IV}^{-}=1$), the error rate would also quickly increase, as *k*_*IV*+_ → 10^4^ s^−1^ (*Err* ~ 10^−2^). These results indicate that even when the rate-limiting conformational transition is not directly involved in the substrate selection, the rate enhancement of this slow step still impacts much on the error rate, such that the transition cannot be too fast or too slow, as noted above.

In summary, since the current parameter setting from T7 RNAP attributes the rate-limiting conformational transition to the NTP insertion step [21] (from the pre-insertion state *III* to the insertion state *IV*) but not the catalytic step (from the insertion state *IV* to the product sate *V*), the elongation error rate is maintained at close-to-minimum under the pre-insertion dominated strategy. Even in an equally mixed selection strategy ($\eta_{III}^{+}=\eta_{III}^{-}=10\;\&\;\eta_{IV}^{+}=\eta_{IV}^{-}=10$), *k*_*IV*+_ ~ 10^3^ s^−1^ still appears to be too large such that the error rate reaches ~ 4 × 10^−3^. Additionally, from the elongation rate plots (see Figure 7 and Figure 8), one can see that the post-insertion selection strategy leads to a quite low elongation speed, which seems unlikely. Hence, current analyses seem to suggest that the pre-insertion dominated selection strategy applies for the T7 RNAP elongation.

## 4. Discussion

In this work, we show how the rate changes at different kinetic steps impact on the overall elongation characteristics, when the stepwise kinetic model of NAC is considered. Note that we address polymerases with high selection accuracies but not those error-prone ones, so that overall, the kinetic discrimination applies [10]. That being said, we focus on analyzing how the non-equilibrium driving force, which enables high fidelity control by promoting fast kinetics, balances with substrate selection activities at the kinetic checkpoint, which require slowing down the corresponding processes.

Previous literature on template-based polymerization has clearly pointed out that high fidelity or low error rate is achieved at far-from-equilibrium [6,7,9], without considering the stepwise kinetics within each polymerization cycle. Nevertheless, in realistic gene replication and transcription processes, the polymerization NAC consists of multiple steps [4,11,12,21], from nucleotide binding, insertion to pre-chemistry adjustments, and then the catalytic reaction, product release, and polymerase translocation. Correspondingly, the selection against non-cognate nucleotide species in each NAC may not necessarily or can hardly be achieved via a single step. Corresponding to the multi-step kinetics within each NAC, there exist variable ways to approach to the far-from-equilibrium. For the purposes of the protein functional determination or structure-function design, one wonders how a polymerase system can be kinetically optimized for efficient elongation and fidelity control. We accordingly show that the kinetic variations at different steps impact differently on the overall elongation performances, primarily depending on whether the step differentiates the nucleotide species or not. 

For forward transitions with no substrate differentiation, such as the nucleotide binding (i.e., the diffusion-limited process) and polymerase translocation steps, acceleration on the transition quickly enables the system to approach to sufficiently far from equilibrium with a converging low error rate. Further acceleration leads to the overall elongation rate saturation. Subject to solution or protein environmental constraints, these steps are supposedly to act quickly in order to allow high polymerization speed and fidelity simultaneously.

In contrast, for the forward transition that differentiates the nucleotide species and serves as the selection checkpoint, i.e., by inhibiting the addition rate of the non-cognate substrate species, the continuous acceleration of such a step leads to an increasing error rate, even though the system is energetically pumped to far-from-equilibrium, with a high elongation speed being obtained. The corresponding entropy production increases but deviates from the heat dissipation or the free energy expenditure for each polymerization cycle, due to an increasing error rate. Hence, strong acceleration at such a selection checkpoint needs to be avoided. On the other hand, one might consider that slowing down the forward transition at the selection checkpoint guarantees the system to gain sufficiently high accuracy, though at the sacrifice of the speed. Yet one sees that by simply slowing down the checkpoint transition to near the equilibrium condition, high error rates also emerge, in particular, as an entropy-driven regime exists close to the equilibrium condition [6]. In brief, the transition at the selection checkpoint needs to proceed not too slowly nor too quickly, in order to achieve a close-to-minimum low error rate. Interestingly, we found that the corresponding nucleotide addition or insertion step in T7 RNAP takes place right at a moderate rate of ~10^2^ s^−1^, which is about optimal for the error reduction. In reality, an enzyme cannot shift the free energy drive or expenditure over a cycle, so that the kinetic modulation for a certain conformational transition must be balanced by modulation(s) for some other internal transition(s) of the enzyme. It is highly desirable to understand how a polymerase enzyme coordinates stepwise kinetics for several internal transitions to achieve an efficient elongation and fidelity control. 

The accelerations on the backward transitions show similar but opposite trends: the accelerations lead to close-to-equilibrium with low speeds and high error rates; slowing down the transitions promotes far-from-equilibrium with high speeds and low error rates, except for the selection checkpoint at which the error rate rises for the too slow backward transition. Correspondingly, the rate for the backward selection also needs to be maintained at a moderate level, not only for the fidelity control, but also to coordinate with subsequent forward selection transition to maintain the overall speed high.

In order to elucidate the above ideas, we employed the three-state and five-state models of the polymerase NAC as in realistic genetic processes [12,17,21]. In particular, we used kinetic parameters that are consistent with biochemical and single molecule force measurements on the representative T7 RNAP system [17,21], which relies on nucleotide selection but has no detectable proofreading for fidelity control. Notably, for kinetic rate parameters such as the forward addition or insertion rate of NTP, and the backward NTP dissociation or the off rate from RNAP, both rates appear to be optimal (~10^2^ s^−1^) for the error reduction, i.e., leading to an error rate as previously suggested (*Err* → 10^−4^) [27]. Such a ‘rate-design’ at the selection checkpoints must rely on some conformational transition features of the protein, that manifest merits obtained in the evolution of the polymerases to act sufficiently accurate and fast for the genetic regulation of the corresponding organisms.

Note that current analyses on the stepwise acceleration or rate modulation are conducted when the selection strengths or the corresponding differentiation energetics remains constant at the selection checkpoints. How to allocate the differentiation or selection energetics, possibly subjecting to a total energy budget, had been examined using the same stepwise kinetic framework [13]. The study essentially draws attention to ‘early’ selections on the reaction path, which take place right after the NTP binding to the polymerase. In current study, we additionally show that the stepwise kinetics matters, and the rate-limiting transition plays a significant role in the error control. The rate-limiting transition often serves for the selection checkpoint that directly controls the error rate, which is likely the reason that the corresponding transition proceeds slowly so that relevant conformational degrees of freedom relax sufficiently well for the nucleotide selection activities. We notice additionally that even when the rate-limiting step does not serve for a selection checkpoint, varying the limiting rate still affects the elongation error rate. Though it is not yet clear which transitions serve for the selection checkpoint exactly in the NAC of T7 RNAP, our analyses suggest that the pre-insertion state can play a dominant role such that both the initial screening upon the NTP per-insertion and the subsequent rate-limiting insertion process are critical for the nucleotide selectivity. Some structure-based computational studies have already been conducted to elucidate the pre-insertion selection of nucleotides in T7 RNAP [22,28,29]. Consistently, it has been detected that in T7 DNAP, the catalytic step is rate-limiting and also serves as a dominant selection checkpoint against non-cognate nucleotide species [12].

Recently, experimental studies have nicely conducted on the T7 RNAP fidelity control by measuring incorporation and mis-incorporation rates for specific nucleotides [23,24]. Nevertheless, to reveal the selection activities throughout the NAC, stepwise kinetics for the cognate and non-cognate nucleotides are still to be determined, as that which was performed for T7 DNAP [12]. Meanwhile, intensive atomistic simulations are currently being conducted to provide the stepwise free energetics of the nucleotide selection in T7 RNAP; in particular, during nucleotide insertion (in preparation). Additionally, incoming studies need to take into consideration the sequence specificities in the nucleotide selection. Besides, the fidelity control also involves base deletion and addition of types of errors due to frameshift [30,31], etc., which we have not yet considered. With systematic experimental and computational approaches, the underlying structural dynamics mechanisms of nucleotide selectivity, along with design principles for the polymerase fidelity control, are expected to be reveal.
